# Supplementary figures and images for: Behavioral adjustment of C. elegans to mechanosensory loss requires intact mechanosensory neurons
Source: PLoS Biol. 2024 Jul 18;22(7):e3002729. doi: 10.1371/journal.pbio.3002729 (PMC11288434; doi:10.1371/journal.pbio.3002729)

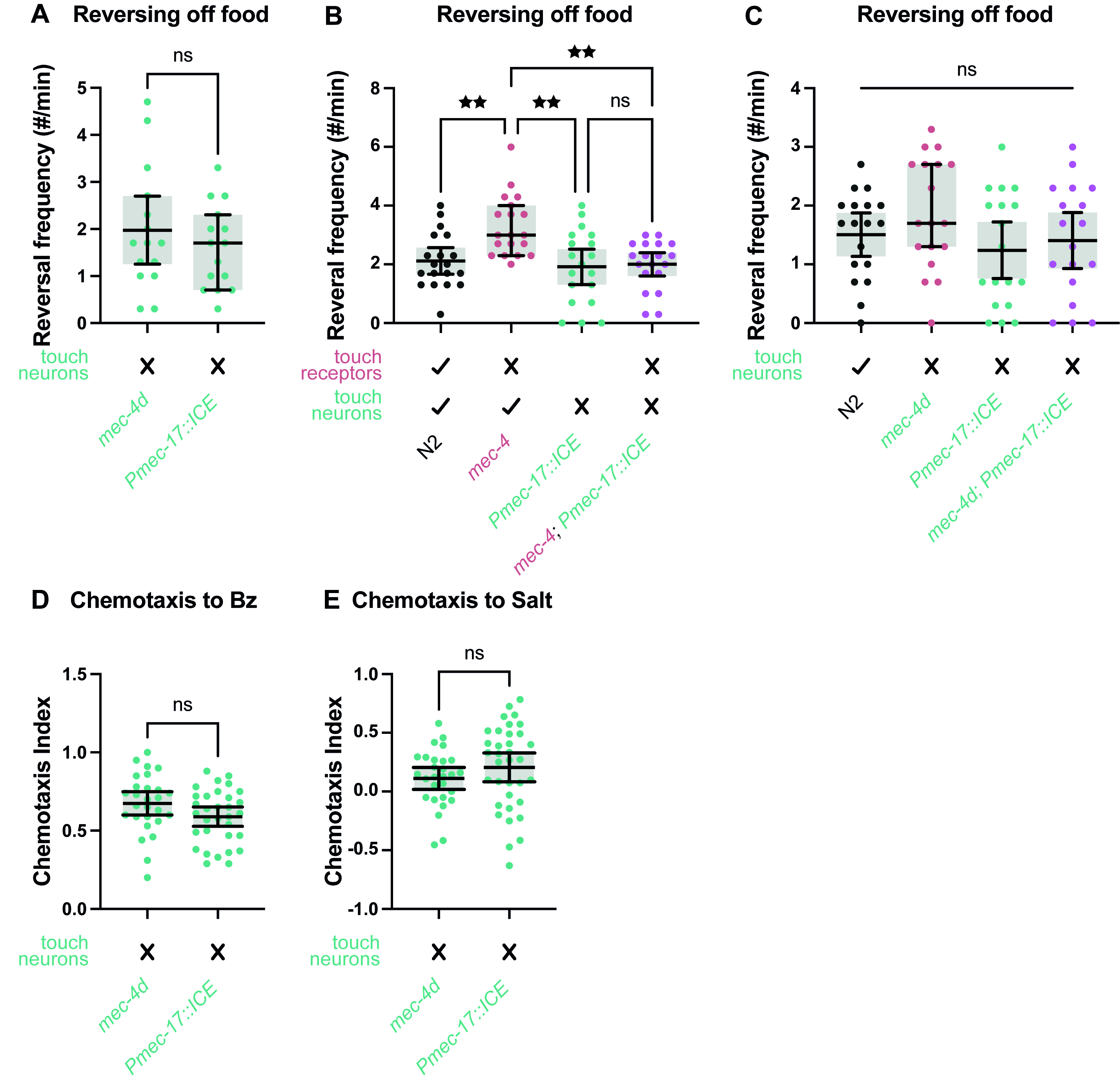

Supplement: S1 Fig — (A–C) Spontaneous reversing frequency in the absence of food. (A) Šídák’s test, n = 15 for each condition. (B) Kruskal–Wallis test (p < 0.0001) followed by Dunn’s multiple comparisons test. ★★p < 0.01. n = 19 for each condition. (C) One-way ANOVA (p = 0.2). n = 18 for each condition. (D) Chemotaxis to 1:1,000 Benzaldehyde (Bz). Dunn’s test, n = 27 for each condition. (E) Chemotaxis to 100 mM NaCl. Dunn’s test, n = 28, 35. The data presented in panels A, D, and E was obtained in the same experiments as shown in Fig 1B–1D, respectively. The numerical data presented in this figure can be found in Supplementary file S1 Data. (TIF) [file pbio.3002729.s002.tif]

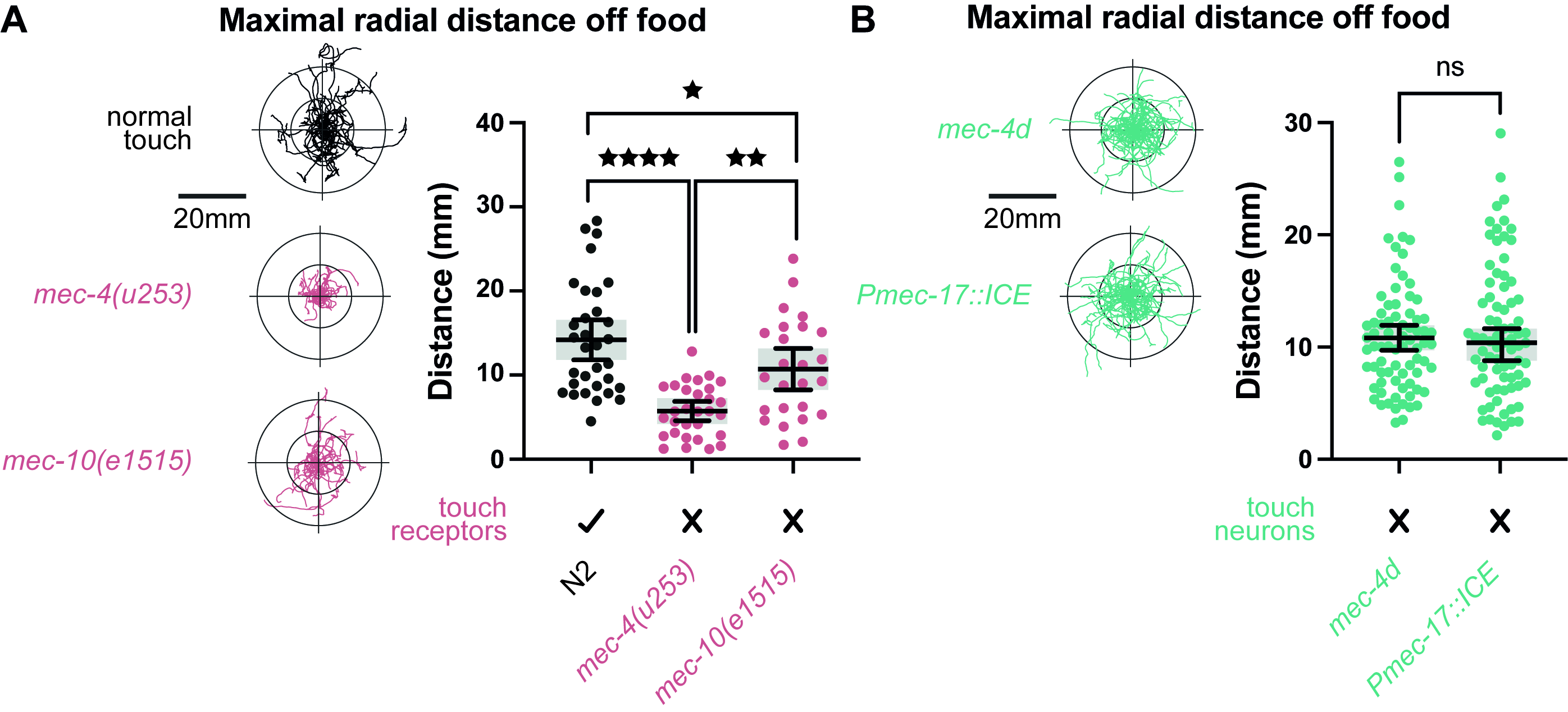

Supplement: S2 Fig — (A, B) Left, aligned worm trajectories in the absence of food over a 2-min time window. Right, maximal radial distance of worm trajectories. (A) One-way ANOVA (p < 0.0001) followed by Tukey’s multiple comparison test. ★p < 0.05, ★★p < 0.01, ★★★★p < 0.0001. n = 32, 30, 25. (B) Dunn’s test, n = 76, 77. The data presented in this plot was obtained in the same experiment as shown in Fig 2A. Bars indicate mean with 95% confidence intervals. The numerical data presented in this figure can be found in Supplementary file S1 Data. (TIF) [file pbio.3002729.s003.tif]

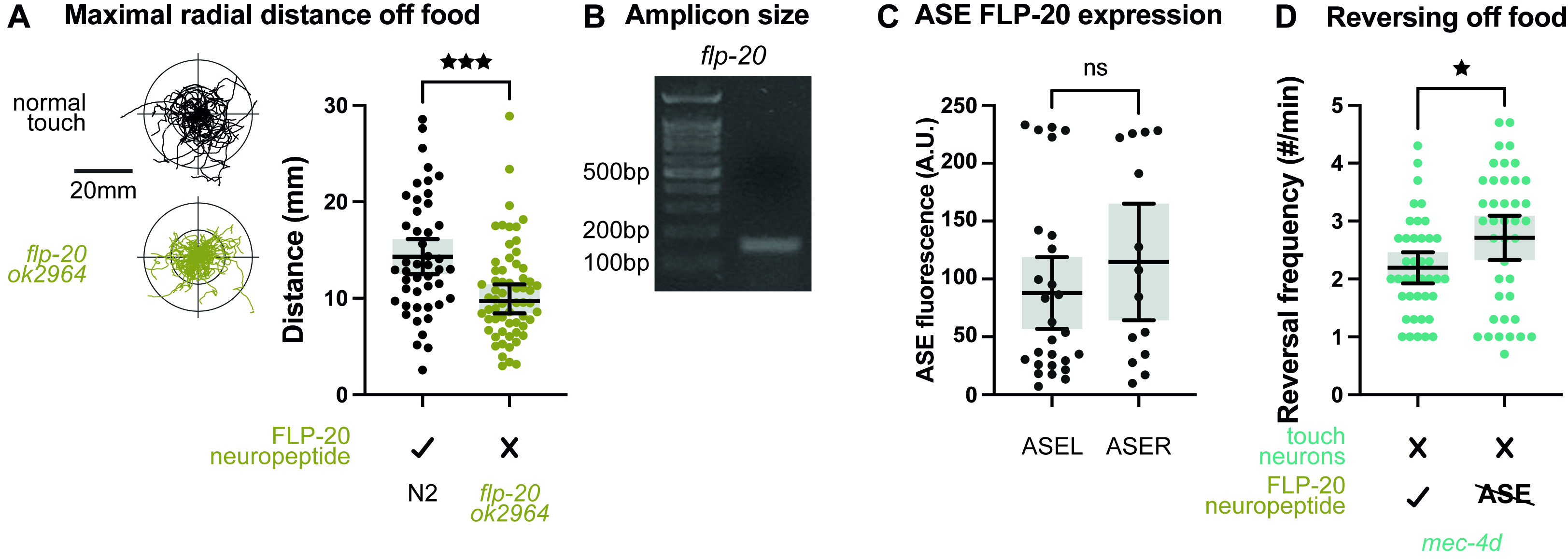

Supplement: S3 Fig — (A) Left, normal vs. flp-20(ok2964) mutant trajectories in the absence of food over a 2-min time window. Right, maximal radial distance from the start point of each trajectory. Mann–Whitney test. ★★★p < 0.001. n = 46,62. (B) flp-20 RT-qPCR primers (5′-TGGTTATCCTGGTCAAGAGC-3′; 3′-TCATGTGGTTCATCTGTGCC-5′) produce an expected specific 152-bp long genomic amplicon. (C) Mean ASEL vs. ASER fluorescence within the ASE cell body after background subtraction. Mann–Whitney test. n = 27, 14. (D) Reversing frequency off food of mec-4d with normal vs. ASE-specific silenced FLP-20. Mann–Whitney test. ★p < 0.05. n = 40. Bars indicate mean with 95% confidence intervals. The numerical data presented in this figure can be found in Supplementary file S1 Data. (TIF) [file pbio.3002729.s004.tif]

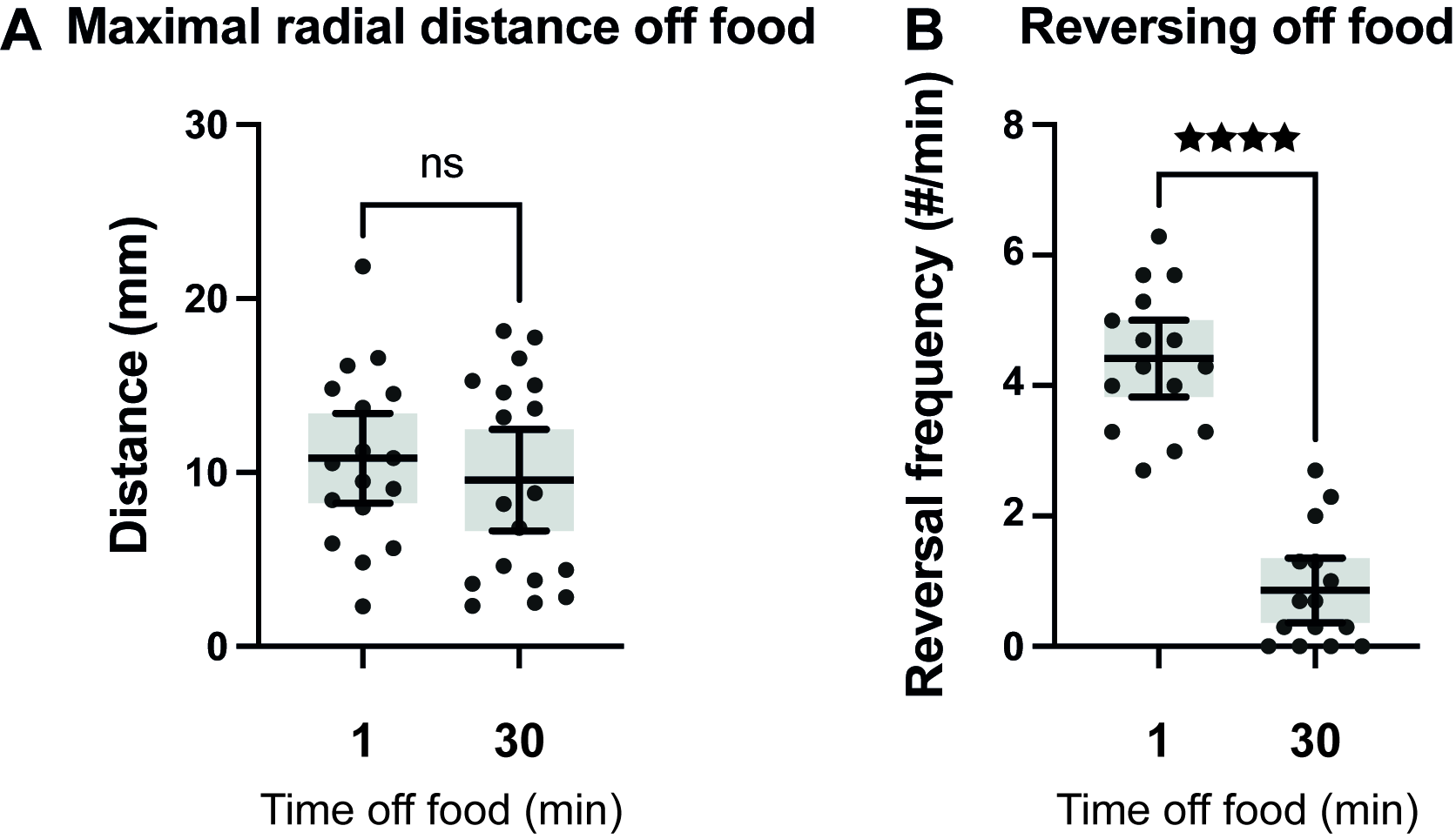

Supplement: S4 Fig — (A, B) Comparison between worms that spent 1 min vs. 30 min off food before the start of the assay. (A) Maximal radial distance from the start point of each trajectory, Mann–Whitney test (p = 0.503). n = 17, 18. (B) Reversing frequency off food, Mann–Whitney test. ★★★★p < 0.0001. n = 15 for each condition. Bars indicate mean with 95% confidence intervals. The numerical data presented in this figure can be found in Supplementary file S1 Data. (TIF) [file pbio.3002729.s005.tif]
