# Supplementary material for: Behavioral adjustment of C. elegans to mechanosensory loss requires intact mechanosensory neurons
Source: PLoS Biol. 2024 Jul 18;22(7):e3002729. doi: 10.1371/journal.pbio.3002729 (PMC11288434; doi:10.1371/journal.pbio.3002729)
Supplement: Attachment — Submitted filename: Staum et al point by point reply to reviewers.docx [file pbio.3002729.s006.docx]

We thank the editors and reviewers for the careful work. Having considered the various comments and suggestions, we would like to submit a revised and improved manuscript, including new data that further support our conclusions. We hope that these address the reviewer concerns. Our responses to each point raised by the reviewers are detailed below in blue, and are embedded as comments in an attached copy of the revised manuscript.

------------------------------------------------------------------------
Reviewer remarks:

Reviewer #1: The authors previously reported that worms insensitive to touch become sensitized to odors, leading to enhanced olfaction. In this paper, the authors extended their previous finding by showing that this enhanced olfaction requires touch receptor neurons. They also found that touch insensitive worms showed additional altered behaviors such as nose touch, reversal distance, head oscillations, etc. Finally, they found that FLP-20 expression in TRN-ablated worms was increased in ASE neurons, which is different from their previous observation in touch insensitive mutants.

The paper is relatively simple and well presented. It is a nice follow-up study of their previous PLoS Bio paper. The authors also put forth an interesting twist by speculating altered behaviors in touch-deficient worms as cautious-like responses.

Thank you. We appreciate this feedback.

On the other hand, I find the work a bit thin and lack of depth.

They did not show how an increased expression of FLP-20 in ASE neurons in TRN-ablated worms led to the observed behavioral phenotypes as done in their previous paper.

Thank you for this comment. To address this, we have now carried out an additional experiment that shows how loss of *flp-20* specifically in the ASE neurons results in behavioral modification even in worms lacking the TRNs. These new results are now reported in Fig. 5B and S3D Fig.

They did not perform functional characterizations of the neurons involved by recording their activities (calcium imaging) as done in their previous paper. These weaknesses dampened my enthusiasm.
To appreciate the differences between loss of touch sensation and loss of the touch neurons at the neuronal level, we decided to focus on the motor neuron/interneuron RIM. This neuron plays opposing roles in regulating reversing and locomotion, and as such, could be associated with the behavioral adjustments following loss of touch. We recorded spontaneous activity from this neuron in control, *mec-4* and *mec-4d* worms, and observed a decrease in the magnitude of RIM calcium events in *mec-4* in comparison to N2, whereas *mec-4d* mutants showed no such change (new Fig. 3). This result is consistent with our behavioral data and with the differences that we have found so far to exist between *mec-4* and *mec-4d* worms. At the same time, we did not observe a clear difference between the average RIM activity in *flp-20* mutants compared to N2 (new Fig. 4D). This could imply that the FLP-20 pathway involves other neurons than RIM and/or that the specific impact of FLP-20 on RIM may in its downstream signaling rather than in its ongoing calcium activity.


Reviewer #2: In the present manuscript Staum and colleagues have compared behavioral impairments in C. elegans nematodes lacking an important mechanosensory channel (mec-4) known to mediate gentle touch-induced reversals with animals where the sensory neurons responsible for this touch response are genetically ablated. They report several differences in terms of behavioral responses and in gene expression between these two distinct conditions and go on to assign an ecological interpretation to the meaning of their findings.
I will begin by saying that the experiments appear expertly conducted, with appropriate sample sizes and statistical tests performed.

Thank you. We appreciate this feedback.

My principal criticism of this work is not in the figures or experiments presented but principally with the interpretation of the results or even the very story weaved together with these findings. For this, I found some serious problems which I will attempt to communicate below.

First, the description of the neurons and genes involved in this study are very vague and incomplete and betray the complexity of the system. A naïve reader might erroneously conclude that mec-4 is only expressed in touch sensitive neurons, or that the touch circuits described are the only ones present in the worm. This would be incorrect. In fact, mec-4 is expressed in close to 50 cells (mostly neurons) involved in many behaviors (for a list see CeNGEN dataset here: [https://cengen.shinyapps.io/CengenApp/](https://cengen.shinyapps.io/CengenApp/)).

We find this somewhat imprecise. *mec-4* is an extensively characterized gene in *C. elegans*, repeatedly shown to express exclusively in the touch neurons (e.g., Mitani et al., 1993; Zhang et al., 2004; Neumann et al, 2011). The CeNGEN dataset provides several levels of filtering and thresholding. The only way to derive from the dataset 48 neurons that express *mec-4* (as suggested by the reviewer) is by removing all filters from all cells (this means a lot of false positives). In contrast, once any thresholding of any degree of stringency is applied, the list immediately shrinks to just the 6 touch receptor neurons (+ CAN, whose function is not clear and is not known to have any chemical synaptic connections with any neuron). The same exercise can be done to any other cell-specific gene. Indeed, the thresholding in CeNGEN is based on a comparison to a subset of ‘ground truth’ expression patterns (which did not include *mec-4*). Just as an example, *ceh-31*, which in reality is expressed in only 6 neurons, when unfiltered, shows expression in 88 neurons in the CeNGEN database. We now emphasize the known specificity of *mec-4* expression in the first subsection of the Results.

How loss of mec-4 in these tissues might affect the different behaviors studied here, its hard to say. The proper way to conduct the experiment could be to perform cell-specific silencing of mec-4 in the mechanosensory neurons known to mediate gentle touch reversals.

Given the restricted expression of *mec-4*, we find this to be unnecessary. Even the approach suggested by the reviewer were to be taken, then it would suffer from circularity, since the reviewer could argue that the TRN-specific promoters themselves are not be exclusive to the touch neurons, making it hard to interpret the results. This was the reason for choosing *mec-4* in the first place, because of its highly restricted expression pattern.

Second, it is not clear how backcrossed the mec-4(u253) strain is. It is standard to backcross these strains at least 5 (but preferably 10) times to remove unwanted background mutations. The strain description of the nematode repository (CGC) does not list the number of crosses. The danger here would be that uncharacterized background mutations could potentially affect some of the behaviors under study (a risk that increases with the number of processes under scrutiny).

These issues have already been addressed in our preceding paper (Rabinowitch et al. 2016), in which an additional touch receptor subunit mutant, *mec-10(e1515)*, was used to confirm the *mec-4(u253)* phenotype. In that paper, we also showed that artificial activation of the touch neurons in the *mec-4(u253)* strain, was sufficient for restoring locomotion to normal, indicating that the **cause** for the locomotion phenotype is directly related to touch neuron activity. Finally, in that previous study, we also demonstrated that removing FLP-20 specifically in the touch neurons resulted in a similar phenotype to that of touch mutants, including *mec-4(u253)*. In the current study we have also shown that eliminating the touch neurons in *mec-4(u253)* abolishes the phenotype (S1B Fig). In any case, as an extra measure, we have now also performed the dispersal assay, the main behavioral test of the current study, using *mec-10(e1515)* mutants (S2A Fig), which produced a similar phenotype as *mec-4(u253)*, although less pronounced, considerably reducing the risk of a background genetic effect.

Another thing I thought was problematic was the lack of mention of other mechanosensory neurons and circuits in the animal. For example, the CEP neurons are mechanorsensory and in the head of the worm. It would make a ton more sense that a compensatory process harnessed other existent mechanosensory neurons to make up for deficits in a different one (more sense than harnessing a different sensory modality altogether).

Actually, we did examine other mechanosensory neurons, namely, the nose touch circuit, and showed modified nose touch responses in body touch-deficient worms (Fig. 2B). We found that loss of body touch, rather than leading to greater sensitivity to nose touch, actually restricted their responses, which we interpret as a possible cautious behavior. As for the CEP neurons, we have already addressed this in a previous paper (Rabinowitch et al., 2016). These neurons are involved in the mechanosensory detection of food, eliciting slowing on food (Sawin et al., 2000). In our previous paper we found no differences in slowing on food between control and touch-deficient worms. Both these results rule out a **compensatory** process harnessing alternative mechanosensory neurons.

On this topic, cross-modal sensory compensation in humans is not a process that occurs at the periphery but centrally. Meaning that cortical processing of sensory information is altered in individuals who suffered some sensory loss. It is not something at the level of the receptor. Blind people do not acquire the ability to see with their fingers or acquire more mechanosesory neurons or even receptors. Rather the weight given to other modalities increases in response to the loss of another modality. Therefore, the premise of this study is flawed in its comparison to this phenomenon in humans.

There are definitely many differences between *C. elegans* and humans. Nevertheless, our previous paper (Rabinowitch et al., 2016) actually showed that the increase in olfactory acuity following mechanosensory loss does not lead to the formation of new mechanosensory neurons or receptors, but rather results in altered synaptic communication within the olfactory circuit between the chemosensory neurons and the interneurons that integrate sensory information, which is more in line with a shift in the weight given to other modalities. Indeed, in that paper we suggested how this model may parallel cross-modal processes that occur also in more complex mammalian systems. In addition, as part of the revision of the current study, we have examined the effects of loss of body touch sensation and loss of body touch neurons on the ongoing activity of the RIM interneuron, which plays an important role in regulating locomotion (the main behavioral feature in the current manuscript). We now show that RIM activity decreases in *mec-4*, but not *mec-4d* mutants (new Fig. 3 within a new subsection, and new Fig. 4D), indicating that the altered behaviors are due to modifications in the integration of sensory information by the interneurons (again, far away from the periphery).

One final point is with regards to measuring fl-20 expression. Both strategies used here are flawed. First of all, fl-20 is expressed in many cells in addition to mec-4 expressing touch neurons. Therefore, when fl-20 levels are being compared between different strains it is impossible to conclude that this is linked to ASE expression. There are ways to do this, people often use cell specific tags or do single-cell RNAseq or qPCR, however this study used whole animals and therefore reported global fl-20 expression levels. Secondly, the ASE::fl-20::GFP strain used to quantify expression of fl-20 in ASE neurons is not using an fl-20 3' UTR to drive expression but rather the unc-54 3'UTR present in the backbone plasmid constructed by Kim and colleagues. This means that GFGP expression is expected to greatly surpass the natural endogenous levels of fl-20 expression in these cells. That means that changes in GFP brightness reported, while correlated, are not equivalent with levels
of fl-20 expression.

We disagree. (1) The RT-qPCR experiment was intended to address whether a global difference exists in FLP-20 expression between *mec-4* and *mec-4d* mutants, even before trying to identify the source of such a difference. For this purpose, a measurement of total FLP-20 expression is valid and, indeed, even underestimates the effect because the background FLP-20 levels from non-involved neurons may mask the more delicate effects of the participating neurons. (2) The purpose of the *Pflp-20::GFP* fluorescence measurements in the ASE neurons was to compare relative ASE FLP-20 expression levels between *mec-4* and *mec-4d* mutants. The intention was to provide a qualitative answer (hence the arbitrary units on the ordinate). We do not consider the results as any quantification of the absolute level of FLP-20 expression. In addition, as part of the revision we have performed new experiments testing the requirement for FLP-20 expression in the ASE neurons for the suppression of loss of touch modifications following loss of the touch neurons. These new results are now presented in the revised manuscript in the new Fig. 5B and new S3D Fig.
