# Supplementary material for: Behavioral adjustment of C. elegans to mechanosensory loss requires intact mechanosensory neurons
Source: PLoS Biol. 2024 Jul 18;22(7):e3002729. doi: 10.1371/journal.pbio.3002729 (PMC11288434; doi:10.1371/journal.pbio.3002729)
Supplement: Attachment — Submitted filename: Rebuttal 2.docx [file pbio.3002729.s008.docx]

We thank the reviewer for a second round of review. Below please find our responses to the reviewer comments.

The authors have included some new data, but they fall short of addressing my major concerns. For example, theyimaged RIM neuron activity, but this is insufficient to explain the observed differences in mec-4 and mec-4d worms.They should image sensory neurons (ASE and AWC) and additional interneurons (AIB, AIY and AVA) in wt, mec-4 andmec-4d worms as they did in their previous paper. For the newly reported "restrictive-like behaviors", they should atleast image ASH neurons that sense nose touch stimuli. In their previous paper, they provided a nice explanation tohow FLP-20 released by TRNs may inhibit AWC-AIY information flow, thereby inhibiting chemotaxis behavior. But thismechanism cannot explain their current observations. So what is the mechanism? Overall, the work is interesting butrather descriptive and superficial and lacks depth. I would like to see the kind of mechanistic study the authorsaccomplished in their previous work published in PLoS Biology in 2016.

The list of experiments suggested in the new review includes a replication of a considerable part of our previous study. However, most of these experiments are not very relevant to our new study. One of the main points from the 2016 paper was that the sensory neurons of normal and touch-deficient worms receive the same information exactly (they show similar calcium responses). It is how this information is transmitted to other interneurons that matters. For this reason, imaging AWC, ASE or ASH sensory neurons will result in similar responses for all conditions, providing little or no information about the difference between loss of touch and loss of touch neurons.

Also, in the previous study, AIB and AVA interneurons were ruled out as conveying cross-modal plasticity, so it is hard to see the benefit of imaging them either.

The only neuron that did change its activity in the 2016 paper was the AIY interneuron, in response to AWC stimulation. However, this was in the context of odor chemotaxis (the AWC is a chemosensory neuron). In the current context of free locomotion and other restrictive-like behaviors, the RIM neuron seems to be a much more relevant player. This was the reason for focusing on RIM activity, and indeed it showed clear differences between normal, touch-deficient and touch neuron-lacking worms. Therefore, imaging a large set of neurons already shown to not be relevant to behavioral adjustment following loss of touch is not likely to advance our understanding of why no behavioral adjustment occurs when the touch neurons are removed.

The reviewer also claims that we are presenting descriptive superficial results. In fact, our study has revealed an important twist on the previously described mechanism for cross-modal plasticity, showing that as long as the touch neurons are present the cross-modal mechanism is valid. However, in the absence of touch neurons a different mechanism comes into play, and a substantial portion of the new study is dedicated to unraveling this new mechanism and showing that with no touch neurons, a separate set of salt-sensing neurons becomes involved, and that the release of FLP-20 from these neurons replaces the normal role of the touch neurons in FLP-20 signaling.

We acknowledge that we haven't fully uncovered how the ASE neurons assume this new role. This is a question for a separate study outside of the scope of the present one. Notwithstanding, our work shows three novel findings: (1) restrictive-like behaviors are a new manifestation of cross-modal plasticity; (2) the touch neurons have a necessary role in bringing about cross-modal plasticity, and can thus signal not only when the worm is being touched or not being touched, but also when it is unable to sense touch in the first place; (3) as mentioned above, the ASE neurons can take over the role of the touch neurons when these are absent and become involved in FLP-20 neuropeptide signaling.

To nevertheless deepen the level of biological/mechanistic insight stemming from our work, we have expanded our study to include an additional hypothesis regarding the differences between *mec-4* and *mec-4d* worms.

In addition to the role of the FLP-20 neuropeptide as a signal transmitted from the touch neurons promoting dispersal behavior, we have examined the possible participation of additional neuropeptides known to be expressed in the touch neurons: NLP-7, FLP-4 and FLP-8.

Initially it seemed like *nlp-7* mutants display an opposite phenotype to *flp-20*, decreasing rather than increasing their spontaneous reversing off food. Unfortunately, our rescue experiment could not establish this. Testing *flp-4* and *flp-8* mutants showed no changes in behavior, ruling out their participation in TRN-induced behavioral adjustment of these neuropeptides. These results point at FLP-20 as a major signal of touch responsiveness.
